# 2-Methoxyestradiol and Hydrogen Peroxide as Promising Biomarkers in Parkinson’s Disease

**DOI:** 10.1007/s12035-023-03575-6

**Published:** 2023-08-17

**Authors:** Paulina Bastian, Lucyna Konieczna, Jarosław Dulski, Agnieszka Daca, Dagmara Jacewicz, Agata Płoska, Narcyz Knap, Jarosław Sławek, Tomasz Bączek, Leszek Kalinowski, Joanna Drzeżdżon, Anna Roszmann, Mariusz Belka, Magdalena Górska-Ponikowska

**Affiliations:** 1 Department of Medical Chemistry, Medical University of Gdansk, 80-210 Gdansk, Poland; 2 Department of Pharmaceutical Chemistry, Medical University of Gdansk, 80-416 Gdansk, Poland; 3 Department of Neurology, Mayo Clinic, Jacksonville, FL USA; 4 Department of Neurological-Psychiatric Nursing, Medical University of Gdansk, 80-211 Gdansk, Poland; 5 Neurology & Stroke Dpt. St. Adalbert Hospital, “Copernicus” Ltd, 80-462 Gdansk, Poland; 6 Department of Pathology and Experimental Rheumatology, Medical University of Gdansk, 80-210 Gdansk, Poland; 7 Department of Environmental Technology, Faculty of Chemistry, University of Gdansk, Wita Stwosza 63, 80-308 Gdansk, Poland; 8 Department of Medical Laboratory Diagnostics—Fahrenheit Biobank BBMRI.pl, Faculty of Pharmacy, Medical University of Gdansk, 80-211 Gdansk, Poland; 9 BioTechMed Centre, Department of Mechanics of Materials and Structures, Gdansk University of Technology, Narutowicza Street 11/12, 80-233 Gdansk, Poland; 10 Department of Biophysics, Institute of Biomaterials and Biomolecular Systems, University of Stuttgart, 70569 Stuttgart, Germany; 11 Euro-Mediterranean Institute of Science and Technology, 90139 Palermo, Italy

**Keywords:** Parkinson’s disease, 2-Methoxestradiol, Hydrogen peroxide, Oxidative stress, Reactive oxygen species, Biomarkers

## Abstract

Estrogens function in numerous physiological processes including controlling brain cell growth and differentiation. 2-Methoxestradiol (2-ME2), a 17β-estradiol (E2) metabolite, is known for its anticancer effects as observed both in vivo and in vitro. 2-ME2 affects all actively dividing cells, including neurons. The study aimed to determine whether 2-ME2 is a potentially cancer-protective or rather neurodegenerative agent in a specific tissue culture model as well as a clinical setup. In this study, 2-ME2 activity was determined in a Parkinson’s disease (PD) in vitro model based on the neuroblastoma SH-SY5Y cell line. The obtained results suggest that 2-ME2 generates nitro-oxidative stress and controls heat shock proteins (HSP), resulting in DNA strand breakage and apoptosis. On the one hand, it may affect intensely dividing cells preventing cancer development; however, on the other hand, this kind of activity within the central nervous system may promote neurodegenerative diseases like PD. Thus, the translational value of 2-ME2’s neurotoxic activity in a PD in vitro model was also investigated. LC–MS/MS technique was used to evaluate estrogens and their derivatives, namely, hydroxy and methoxyestrogens, in PD patients’ blood, whereas the stopped-flow method was used to assess hydrogen peroxide (H_2_O_2_) levels. Methoxyestrogens and H_2_O_2_ levels were increased in patients’ blood as compared to control subjects, but hydoxyestrogens were simultaneously decreased. From the above, we suggest that the determination of plasma levels of methoxyestrogens and H_2_O_2_ may be a novel PD biomarker. The presented research is the subject of the pending patent application “The use of hydrogen peroxide and 17β-estradiol and its metabolites as biomarkers in the diagnosis of neurodegenerative diseases,” no. P.441360.

**Supplementary Information:**

The online version contains supplementary material available at 10.1007/s12035-023-03575-6.

## Introduction

Estrogens are sex hormones that are endogenously generated from cholesterol and serve important roles in a variety of physiological processes. Different organs and tissues, including the ovaries, testes, adipose tissue, and adrenal cortex, physiologically produce three major forms of estrogen: estrone (E1), estradiol (E2), and estriol (E3). They are known to elicit numerous neuroprotective effects by acting as antioxidants, mostly through estrogen receptors, and so up-regulating the production of antioxidant enzymes, as well as encouraging DNA repair, boosting growth factor expression, and regulating cerebral blood flow. Furthermore, estrogen-dependent signaling pathways regulate neurogenic processes by balancing the proliferation and differentiation of brain stem/progenitor cells. [1]. Likewise, cortical and hippocampal cells have both traditional (ERα and ERβ) and non-classical (GPER-1) estrogen receptors [2–4].

2-Methoxyestradiol (2-ME2), the major physiological metabolite of E2, inhibits cancerous and metastatic processes (breast cancer, pancreatic cancer, Ewing’s sarcoma, and osteosarcoma) by affecting the growth and death mode of several neoplastic cell types both in vivo and in vitro [5–7]. 2-ME2 is present in both men and women and its level in the blood plasma ranges from mere pg/mL in men up to over 10,000 pg/mL in pregnant women [8]. A large number of studies revealed that 2-ME2 selectively induces neuronal nitric oxide synthase (nNOS) in both cancer and neuronal cell lines, notably, at pharmacological and physiological concentrations [9–11]. From a molecular standpoint, 2-ME2 enhances the localization of nNOS in the cell nucleus, resulting in DNA damage from nitro-oxidative stress, which leads to cell cycle arrest and apoptosis in osteosarcoma 143b cells [9–14]. The activation of nNOS and further release of nitric oxide (NO) as observed at physiological levels of 2-ME2, show that 2-ME2 is not just a waste metabolic product but rather a biologically active molecule, and specifically an independent hormone acting on its own [10]. In addition, it is important to note that the above-mentioned mechanism of action of 2-ME2 is not confined to neoplastic cells, but rather to any actively dividing cells, including neurons [15] which is worth noticing in light of the fact that there are actually two sites of active neurogenesis in the adult brain—the dentate gyrus of the hippocampus and the subventricular part of the olfactory bulb [16]. Therefore, it is worthwhile to investigate if 2-ME2 as a potential anti-cancer drug may harmfully affect the brain cells. It has been shown that only pharmacological concentrations of 2-ME2 are also cytotoxic toward immortalized mouse hippocampal HT22 cells, which is an interesting and challenging research prospect [11].

Up to this date, there is no data about the induction of Parkinson-like symptoms in animals by 2-ME2. Several canine cancer cell lines [17], as well as tumor xenograft development nude mice, have been used to research 2-ME2’s anticancer features [18, 19]. In this project, the inverse relationship between PD and cancer incidence has specifically drawn the researchers’ attention. PD patients show some resistance to cancer, even tobacco-dependent cancers [20, 21]. The hippocampus is well known to be undergoing neurogenesis, so it is only logical that it may be particularly vulnerable to the potentially cytotoxic effects of 2-ME2. In addition to that, 2-ME2 is formed from E2 with the involvement of catechol-O-methyltransferase (COMT), an enzyme present in the hippocampus, which supports the claim that 2-ME2 levels may be quite meaningful in that area of the brain [16, 22, 23]. One of the key aims of our research was to clarify whether 2-ME2 can be treated as a physiological factor protecting against cancer induction, and simultaneously the one contributing to the development of neurodegenerative diseases, specifically PD.

In the present study, 2-ME2 effect on the neuroblastoma (NB) SH-SY5Y cell line, as an in vitro PD model, was examined. The obtained results suggest that 2-ME2 generates nitro-oxidative stress and also regulates heat shock proteins (HSP) in NB SH-SY5Y cells leading to DNA strand breaks and consequently resulting in apoptosis.

Parkinson’s disease (PD) is a relatively frequent progressive neurodegenerative disease characterized by a variety of motor and non-motor symptoms [24–26]. Most motor symptoms may manifest at a late stage when the majority of dopaminergic neurons have already been destroyed [27]. Reliable diagnostic and prognostic biomarkers are urgently required for the early diagnosis and possible subsequent treatment of PD at the initial stage.

The other part of our study was carried out in order to compare the results of the in vitro results with our observations based on the clinical model. The concentration of estrogens and their selected derivatives was determined in the plasma of patients presenting with PD by the LC–MS/MS method, and the concentration of hydrogen peroxide (H_2_O_2_) as a powerful biological oxidizer, was determined using our research team's original analytical stopped-flow technique, specifically adopted for the purpose [28–30]. Interestingly, the concentration of H_2_O_2_ as measured in the blood of patients suffering from PD was significantly higher than in healthy individuals. Based on the above, we suggest that plasma levels of hydroxy- or methoxyestrogens, as well as plasma H_2_O_2_ levels may be used in combination as reliable biomarkers for the diagnosis and monitoring of PD. The research results presented in our study are being evaluated as a patent application, entitled: “Use of hydrogen peroxide, and 17β-estradiol and its metabolites as biomarkers in the diagnosis of neurodegenerative diseases,” number P.441360.

## Material and Methods

### In Vitro Studies

#### Cell Culture

Experiments employed NB SH-SY5Y cell line as the neurodegenerative cellular model. Sigma Aldrich (Poznan, Poland) provided human NB SH-SY5Y. The cells were grown in DMEM/F12 medium with 10% FBS, 1% L-glutamine, 1% non-essential amino acids (Sigma-Aldrich), and 1% penicillin/streptomycin at 37 °C with 5% CO_2_.

#### Cell Treatment

First, NB SH-SY5Y cells were seeded in culture media at appropriate densities 24 h before treatment. Next, the cells were treated with physiological (100 pM, 1 nM, and 10 nM) and pharmacological (100 nM, 1 μM, and 10 μM) concentrations of 2-ME2. The investigations were carried out in a medium without FBS in order to completely exclude the influence of hormones derived from sera. The solvent used to prepare 2-ME2 solutions, DMSO (dimethyl sulfoxide, D2438, Sigma Aldrich, Poland), was provided to control cells in the same ratio. The final DMSO concentration in the incubation medium was less than 0.1%.

#### Cell Viability/Cell Proliferation Assay (MTT Assay)

10,000 SH-SY5Y cells per well were seeded in 96-well plates. After 24 h, the cells were treated with 100 pM–10 μM 2-ME2 for the next 24 h. Control cells were solvent-treated and considered as 100% viability. After incubation, 0.5 mg/mL MTT was added (Sigma-Aldrich, Poland). After 4 h at 37 °C, the plates were centrifuged (700 g for 10 min) to remove the supernatant. The formazan crystals were dissolved with 100 μL DMSO (Sigma-Aldrich, Poland). Microplate reader was used to read 570nm absorbance (BioTek Instruments, Inc., USA). The data was presented as a percentage of the control. Each experiment was repeated at least 3 times.

#### Assessment of Cell Death Induction

Flow cytometry was used to measure the level of apoptosis and necrosis. SH-SY5Y cells in the number of 300,000 were seeded into 6-well plates per well. After 24 h, the cells were incubated with 100 pM–10 µM 2-ME2. After trypsinization, cells were centrifuged at 1200 g for 7 min and washed 3 times with ice-cold PBS (Sigma-Aldrich, Poznań, Poland). The cells were treated with Annexin V and Propidium Iodide (PI) for 15 min at room temperature (559763, PE Annexin V Apoptosis Detection Kit I, BD Biosciences). Except for annexin V and PI incubation, the treatment was performed on ice. Annexin V and PI conjugate fluorescence signals were detected with a BD FACSVerse flow cytometer (Becton–Dickinson, Franklin Lakes, NJ, USA). The FlowJo 10.6.1 was used to evaluate the results (FlowJo LCC, Becton Dickinson, Oregon, USA). At least three repeats of the technique were performed to secure repeatability.

#### Assessment of Cell Cycle Arrest

Cell cycle analysis was conducted by flow cytometry. SH-SY5Y cells in the number of 300,000 were seeded into 6-well plates per well. After 24 h, the cells were treated with 100 pM–10 µM 2-ME2. After trypsinization, cells were centrifuged for 7 min at 1200 g. The samples were washed in ice-cold PBS and fixed in ice-cold 70% ethanol overnight at 4 °C. Then, 7 min of 1200 g centrifugation followed. The DNA was stained with 5 g RNase A (E1350-02, EURX, Poland). The final step involved the addition of 10 µg PI (51-66211E, BD Biosciences). FlowJo 10.6.1 was used to evaluate the results. At least three repeats of the technique were performed to secure repeatability.

#### Assessment of ROS Generation

The SH-SY5Y cells in the number of 300,000 were seeded into 6-well plates per well. Next, 100 pM–10 µM 2-ME2 was added to the cells for 8 h. 2′,7′-dichlorofluorescin diacetate (DCF DA, D6883, Sigma-Aldrich, Poland) was added at a final concentration of 10 µM 30 min before the end of incubation. Trypsin was used to remove cells from plates before centrifugation (1200 g for 5 min). The cells were washed twice with PBS, suspended in PBS, and examined by flow cytometry. The procedure was carried out on ice. Flow cytometry counted and analyzed 30,000 cells (BD FACSVerse). FlowJo 10.6.1 was used to evaluate the results. At least three procedures were repeated to guarantee reproducibility.

#### Assessment of RNS Generation

The SH-SY5Y cells in the number of 300,000 were seeded into 6-well plates per well. Next, 100 pM–10 µM 2-ME2 was added to the cells for 8 h. 2′,7′-difluorescein diacetate (DAF-FM DA, D2321, Sigma-Aldrich, Poland) was added at a final concentration of 10 µM 30 min before the end of incubation. Trypsin was used to remove cells from plates before centrifuging (1200 g for 5 min). The cells were washed twice with PBS, suspended in PBS, and examined by flow cytometry. The procedure was carried out on ice. Flow cytometry counted and analyzed 30,000 cells (BD FACSVerse). FlowJo 10.6.1 was used to evaluate the results. At least three procedures were repeated to guarantee reproducibility.

#### Western Blot Analysis

The levels of neuronal nitric oxide synthase (nNOS, ab5583, Abcam, United Kingdom), endothelial nitric oxide synthase (eNOS, ab66127, Abcam, United Kingdom), inducible nitric oxide synthase (iNOS, ab15323, Abcam, United Kingdom), heat shock proteins 60 (HSP 60, sc-13115, Santa Cruz, Dallas, Texas, U.S.A.), and 90 (HSP 90, ab80159Abcam, Great Britain), as well as cytochrome C (sc-13156, Santa Cruz, Dallas, Texas, USA), were determined. After 24 h, at 80% confluence, the cells were treated with 2-ME2 at 100 pM to 10 µM concentrations for further 24 h. The whole Western blot procedure was carried out as previously described [31]. The signal was evaluated using ImageQuant LAS 500 (GE Healthcare, Poland). Densitometry analysis with Quantity One 4.6.7 was used to compute protein levels. The data have been normalized against β-actin. Each experiment was carried out at the minimum of three times.

#### Confocal Microscopy Indicates DNA Strand Breaks

During apoptosis, DNA fragmentation occurs within the nucleus. Using the dUTP end-labeling (TUNEL (terminal deoxynucleotidyl transferase-mediated d-UTP Nick end-labeling)) technique based on the activity of terminal deoxynucleotidyl transferase (TdT), DNA cleavage in apoptotic cells can be identified in situ in fixed cells. TUNEL is a highly specialized method for identifying apoptotic cells. The TdT enzyme catalyzes the addition of labeled dUTP to the 3′ ends of cleaved DNA fragments in the TUNEL assay. Labeled nucleotides: digoxigenin-dUTP or biotin-dUTP can be then identified using confocal microscopy with dUTP coupled to a fluorescent dye.

300,000 SH-SY5Y cells were seeded per well on 6-well plates with circular glass coverslips. The cells were subjected to 24-h-2-ME2 treatments at concentrations between 100 pM and 10 M. Using the TUNEL Andy FluorTM 488 Apoptosis Detection Kit, the TUNEL assay was conducted in accordance with the manufacturer's instructions (A050, ABP Biosciences, USA), as previously described [31]. Confocal microscope images of the cells were captured digitally (Opera PhenixTM, Perkin-Elmer, MA, USA). Both Harmony (Perkin-Elmer, Massachusetts, United States) and ImageJ (v1.52; NIH, United States) were applied for picture processing and merging. Normalized relative fluorescence unit (RFU) to control ratio was used to compute the results and prepare for statistical analysis.

### Clinical Studies

#### Patients’ Biological Material Collection

The studied group consisted of 16 patients diagnosed with sporadic Parkinson’s disease (8 women and 8 men, whose average age was 63 years (± 10), average disease duration 12 years (± 3), average Hoehn–Yahr (H&Y) 2.3 score (± 0.39)) who fulfilled the following inclusion criteria: confirmed diagnosis (according United Kingdom Parkinson’s Disease Society Brain Bank criteria (UKPDS BB) of previously untreated PD stage H-Y I-II, aged 45–70 years.

The healthy controls were 4 women and 5 men, with an average age of 56 (± 10) and no disease data from the history, nor symptoms in the clinical examination and in the brain imaging examination suggesting a diagnosis of symptomatic or atypical parkinsonism.

Both groups were matched for average age and sex. In the control group, any co-morbidities were excluded.

A sample of 20 mL of blood was collected from each patient diagnosed with PD after prior consent to the study. The blood was then centrifuged at 1200 rpm for 10 min to separate the plasma from the red cells. The obtained plasma was used for investigation of E2 metabolites and oxidative stress markers. The red blood cells were disposed. The concentration of estrogens and their selected derivatives was determined in the plasma of PD patients by the LC–MS/MS method, and the concentration of H_2_O_2_ was determined by the proprietary stopped-flow method.

This study was approved by the Independent Bioethics Committee for Scientific Research at Medical University of Gdańsk with the approval number of 195/2020.

#### LC–MS Analysis of Estradiol Derivative Levels in the Blood of Patients Presenting with PD and Control Subjects

The tested plasma samples were obtained from healthy volunteers, participants of a research project carried out by the Department of Neurology and Stroke, The St. Wojciech Hospital, Gdańsk, in cooperation with the Department of Medical Chemistry, Medical University of Gdańsk. All participants in the study provided their written consent.

The concentrations of estrogens (estron (E1) and estradiol (E2)) and their major biological derivatives: 2-hydroxyestrone (2-OH-E1), 2-methoxyestrone (2-ME1), 2-hydroxyestradiol (2-OH-E2), and 2-methoxyestradiol (2-ME2) were examined by liquid chromatography combined with tandem mass detection–LC–MS/MS as previously described [32].

#### Detection of Hydrogen Peroxide (H_2_O_2_) as an Oxidative Stress Marker

After blood collection and separation of the red blood cells, the plasma was collected and centrifuged (200 × g, for 5 min). The cell pellets were washed twice with PBS and then were resuspended in 3 mL of extraction buffer (150 mM NaCl, 5 mM EDTA, 1% Triton X-100, 10 mM Tris–HCl pH 7.4). Insoluble cellular debris was pelleted by centrifugation (500 × g, for 10 min). Supernatants were then analyzed by a stopped-flow technique. The concentration of H_2_O_2_ was determined by the stopped-flow method as previously described [28–30]. For this purpose, a carbon dioxide (CO_2_) biosensor, previously synthesized by our research team, was used—the coordination compound of chromium (III) with pyridoxamine (cis-[Cr (C_2_O_4_) (pm) (OH_2_) _2_] ^+^). In short, the method for H_2_O_2_ determination is based on the assumption of a selective reaction of α-ketoacid—pyruvate with H_2_O_2_ with subsequent decarboxylation of the formed intermediate— pyruvic peracid. The released CO_2_ is being further trapped by the biosensor—cis-[Cr (C_2_O_4_) (pm) (OH_2_) _2_]^+^. The rate of CO_2_ uptake is measured by the spectrophotometric stopped-flow method allowing for determination of the original level of H_2_O_2_ having been converted into CO_2_ in a 1: 1 molar ratio. The reagent solutions were placed separately in two working syringes A and B, respectively: in syringe A—the biological material (sample) and a 5-mM solution of potassium pyruvate mixed in a 1: 1 molar ratio, while in syringe B—1 mM biosensor suspended in a phosphate buffer solution at pH 7.4. Then, the solutions were passed through the mixer (mixing occurs very quickly—3–10 s), and successively through the measuring cell back to the return syringe B. Filling the return syringe with the solution caused the plunger to be pushed against the microswitch. At this point, the flow was stopped and the measurement started. The progress of the reaction in the portion of the solution retained in the measuring cell was monitored spectrophotometrically. During the measurement, the change in substrate concentration was measured as a function of time.

The values of hydrogen peroxide concentrations were for this purpose calculated using the global analysis approach based on collecting the results in the form of a set of absorption spectra that were measured for the entire (specific for a given compound) wavelength range (330–700 nm), in a specified time interval.

#### Kinetic Analysis

Determination of chemical rate constants was carried out by a stopped-flow technique using the Applied Photophysics SX-17MV spectrophotometer. The observable rate constants were computed with “Glint” software. A global analysis of the data acquired for 37 wavelengths, within the range of 340–700 nm at 10-nm increments, was performed for different reaction models.

#### Statistical Analysis of the Results

Statistical analysis was performed using the IBM SPSS Statistics 25 package.

In the case of comparing two groups of people (PD patients and healthy control), the Mann–Whitney *U* test was used. The following descriptive statistics were used in the statistical analysis: median, minimum, maximum, first, and third quartile.

The *p*-value < 0.05 was adopted as statistically significant.

The in vitro results are presented as the mean and standard deviation (SD) calculated from at least three independent experiments. The differences between control samples and 2-ME2-treated samples were assessed utilizing one-way analysis of variance (ANOVA) and Dunnett’s multiple comparison post hoc test. A *p*-value of 0.05 was defined as statistically significant. The GraphPad Prism software was utilized for data analysis (GraphPad Software, Inc., version 8, USA).

## Results

### In Vitro Results

#### Effect of 2-ME2 on Cell Viability

In order to elucidate the mechanism of action of 2-ME2 on NB SH-SY5Y cells, a series of experiments were carried out focusing on 2-ME2 cytotoxicity impact relative to either pharmacological or physiological concentrations of the compound.

Precisely, cytotoxicity of 2-ME2 was determined after treatment of NB SH-SY5Y cells for 24 h with pharmacological (10 µM, 1 µM, and 100 nM) or physiological (10 nM, 1 nM, and 100 pM) concentrations of 2-ME2. Cell viability was determined by the microplate MTT spectrophotometric method. The percentage of viable cells in the sample was calculated as compared to the control cells referred to as 100% viable.

**Fig. 1:**
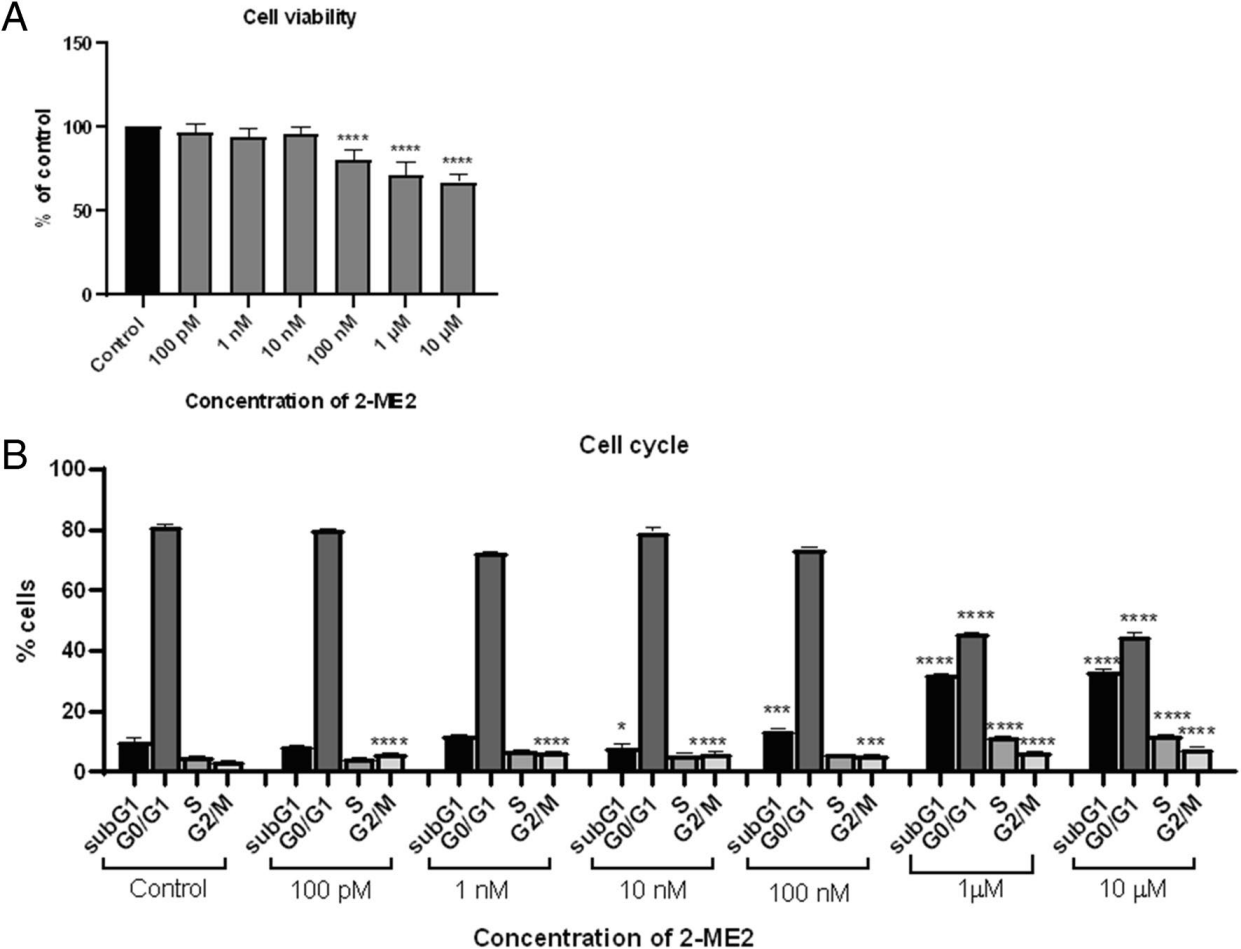
Cell viability and cell cycle after 24h incubation with 2-ME2. **A** Viability of NB SH-SY5Y cells after 24-h incubation with 2-ME2: 2-ME2 was added in a concentration range of 100 pM–10 µM and incubated for 24 h. Cells were harvested and viability was determined by MTT assay. ****p < 0.0001 vs. control. **B** Phases of the cell cycle of 24 h 2-ME2-treated cells NB SH-SY5Y as examined by flow cytometry. *p < 0.1, ***p < 0.001, and ****p < 0.0001 vs. control

Pharmacological concentrations of 2-ME2 inhibited the growth of SH-SY5Y cells by 33% (± 5.17%) for 10 µM, 29% (± 7.53%) for 1 µM, and 19% (± 6.15%) for 100nM, while the physiological concentrations of 2-ME2 (10 nM, 1 nM, and 100 pM) did not seem to reduce cell viability in a statistically significant manner (Fig. 1a).

#### Cell Cycle Analysis After Treatment of 2-ME2 Cells

In the next stage of our research, the influence of 2-ME2 on the NB SH-SY5Y cell cycle was assessed. The NB SH-SY5Y cells were treated with concentrations corresponding to pharmacological (10 µM, 1 µM, and 100nM) or physiological (10 nM, 1 nM, and 100pM) concentrations of 2-ME2 for 24 h (Fig. 1b). Subsequently, the distribution of cells at individual phases of the cell cycle was determined by flow cytometry using the PI dye.

In the case of NB SH-SY5Y cells treated with pharmacological concentrations of 2-ME2, a statistically significant increase in the number of cells in the subG1 phase was observed. The number of subG1-phased SH-SY5Y neuroblastoma cells significantly increased after treatment with 10 µM, 1 µM, 100nM, and also 10 nM 2-ME2, respectively, up to 32.93% ± 0.97%, 32.13% ± 0.39%, 13.66% ± 0.45%, and 7.82% ± 0.99% relative to control (9.99% ± 1.06%). At physiological concentrations, no statistically significant changes were observed except for 10 nM (see above).

As for the G0/G1 phase, a statistically significant decrease in the number of SH-SY5Y neuroblastoma cells was observed after treatment with 10 µM and 1 µM 2-ME2, down to 44.70% ± 1.20% and 45.77% ± 0.38%, respectively, as compared to the control (81.03% ± 0.82%).

In terms of the S phase, a statistically significant increase in the number of SH-SY5Y neuroblastoma cells was observed after treatment with 10 µM and 1 µM 2-ME2 up to 11.90% ± 0.14% and 11.76% ± 0.19%, respectively, as compared to the control (4.91% ± 0.13%).

There was a statistically significant increase in the number of SH-SY5Y neuroblastoma cells in the phase G2/M of the cell cycle as compared to control cells for all 2-ME2 concentrations used. The following values were recorded for respective 2-ME2 concentrations: 7.66% ± 0.39% for 10 µM, 6.58% ± 0.25% for 1 µM, 6.04% ± 0.64% for 100 nM, 6.57% ± 0.30% for 1 nM, and 6.03% ± 0.04% for 100pM relative to control (3.68% ± 0.11%).

#### Analysis of Apoptosis and Necrosis by Flow Cytometry

Subsequently, the induction of apoptosis and necrosis in the NB SH-SY5Y cell line after incubation with 2-ME2 was assessed by flow cytometry technique using annexin V and propidium iodide (PI).

NB SH-SY5Y cells were treated with 2-ME2 at a concentration range from 100 pM to 10 µM for 24 h, and after that time, the number of annexin V-positive and PI-positive cells was measured by flow cytometry method.

**Fig. 2:**
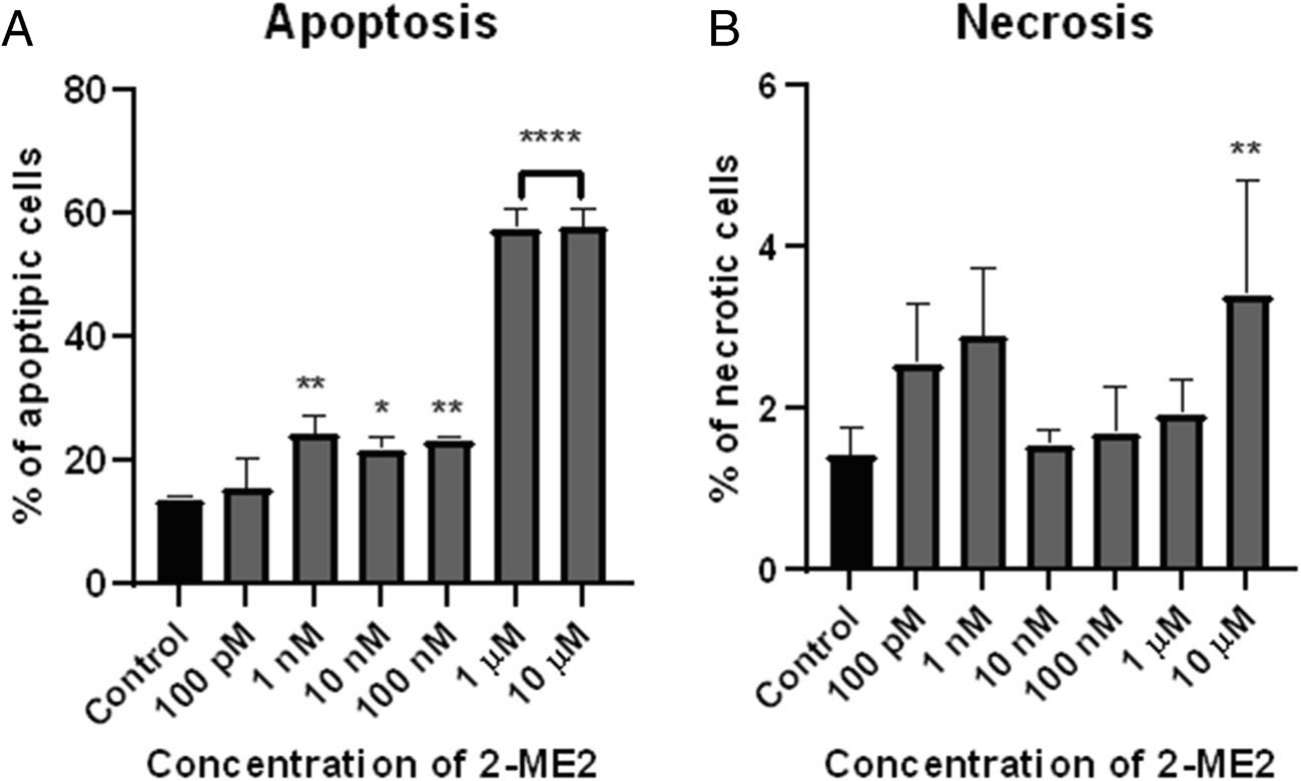
The percentage of cells undergoing apoptosis (**A**) and necrosis (**B**) after 24-h incubation of NB SH-SY5Y cells with 2-ME2 at a concentration range from 100 pM to 10 µM. Values are presented as the mean ± SE from three independent experiments. Data were analyzed with GraphPad Prism Software version 8.0.1 using bidirectional ANOVA with Dunnett's multiple comparison tests against control (*p < 0.1, ^**^p < 0.01, and **** *p* < 0.0001)

The number of apoptotic NB SH-SY5Y cells significantly increased up to 24.27% (± 3.09%) for samples treated with 1nM, up to 21.73% (± 2.14%) for 10 nM, up to 23.3% (± 0.4%) for 100 nM, up to 57.42% (± 3.14%) for 1µM, and up to 57.62% (± 3.11%) 10 µM in comparison to the control with 13.53% (± 0.51%) of apoptotic cells (Fig. 2a). Interestingly, only 10 µM 2-ME2 significantly induced necrosis as observed in 3.85% (± 1.33%) of the treated cells in comparison to the control (1.41% (± 0.35%)) (Fig. 2b).

#### Analysis of ROS and RNS by Flow Cytometry

Induction of nitro-oxidative stress in treated cells is a potential mechanism and consequently the reason for cytotoxic and cytostatic activity of 2-ME2. Thus, in the next stage of our research, intracellular levels of reactive oxygen species (ROS) and reactive nitrogen species (RNS) were examined. Flow cytometry was utilized to carry out the measurements. Cells were treated with 2-ME2 for only 6 h, because of a labile nature and relatively dynamic profile of both ROS and RNS [33].

**Fig. 3:**
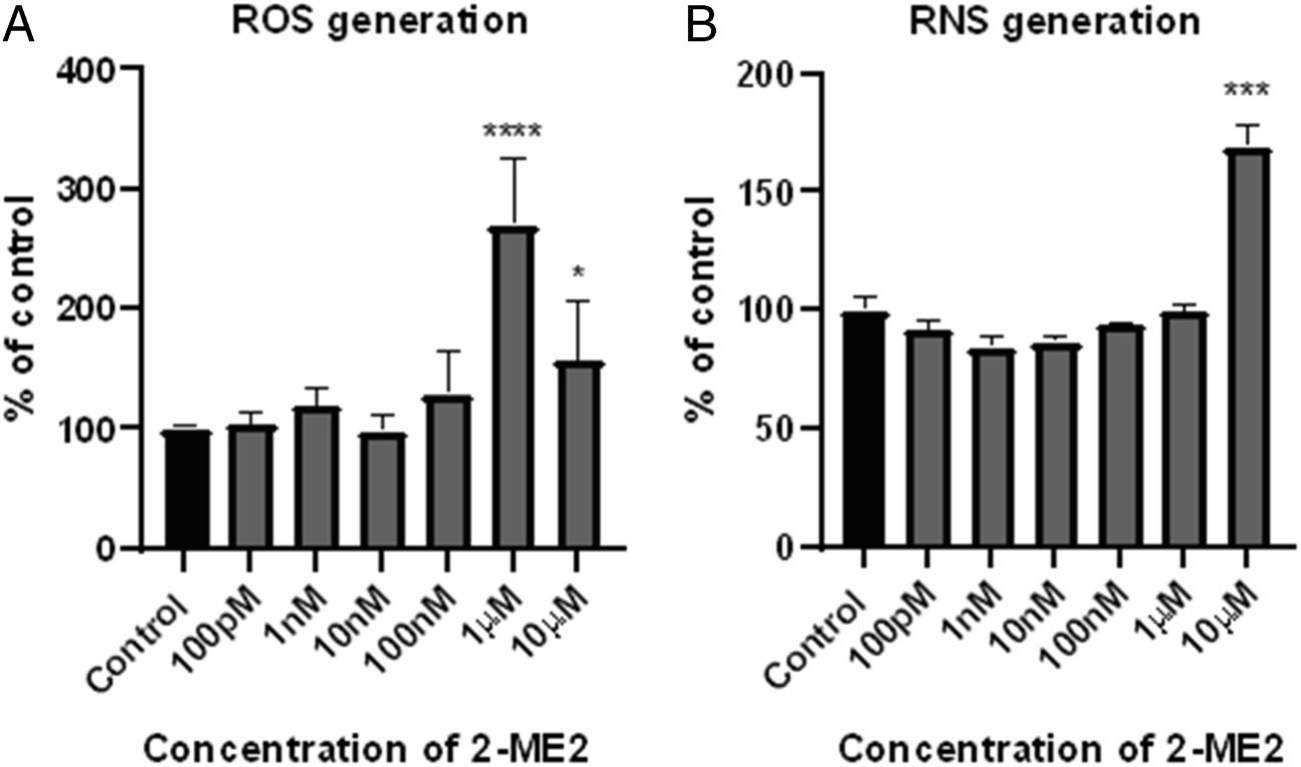
Induction of ROS (**A**) and RNS (**B**) after 6h incubation of NB SH-SY5Y cells with 2-ME2 at a concentration range from 100 pM to 10 µM. Values are the mean ± SE calculated from at least three independent experiments, and expressed as a percentage of the control. Data were analyzed with GraphPad Prism Software version 8.0.1 using one-way ANOVA with the following Dunnett’s multiple comparison tests against control (*p < 0.1, ***p < 0.001, and ****p < 0.0001)

Interestingly, treatment of NB SHSY-5Y cells with 2-ME2 increased the level of ROS up to 270% (± 57%) at 1 µM 2-ME2, and up to 150% (± 50%) 10 µM concentration (Fig. 3), whereas 10 µM 2-ME2 induced RNS generation up to 169% (± 9%) vs. control (Fig. 3b).

#### Effect of 2-ME2 on DNA Fragmentation

**Fig. 4:**
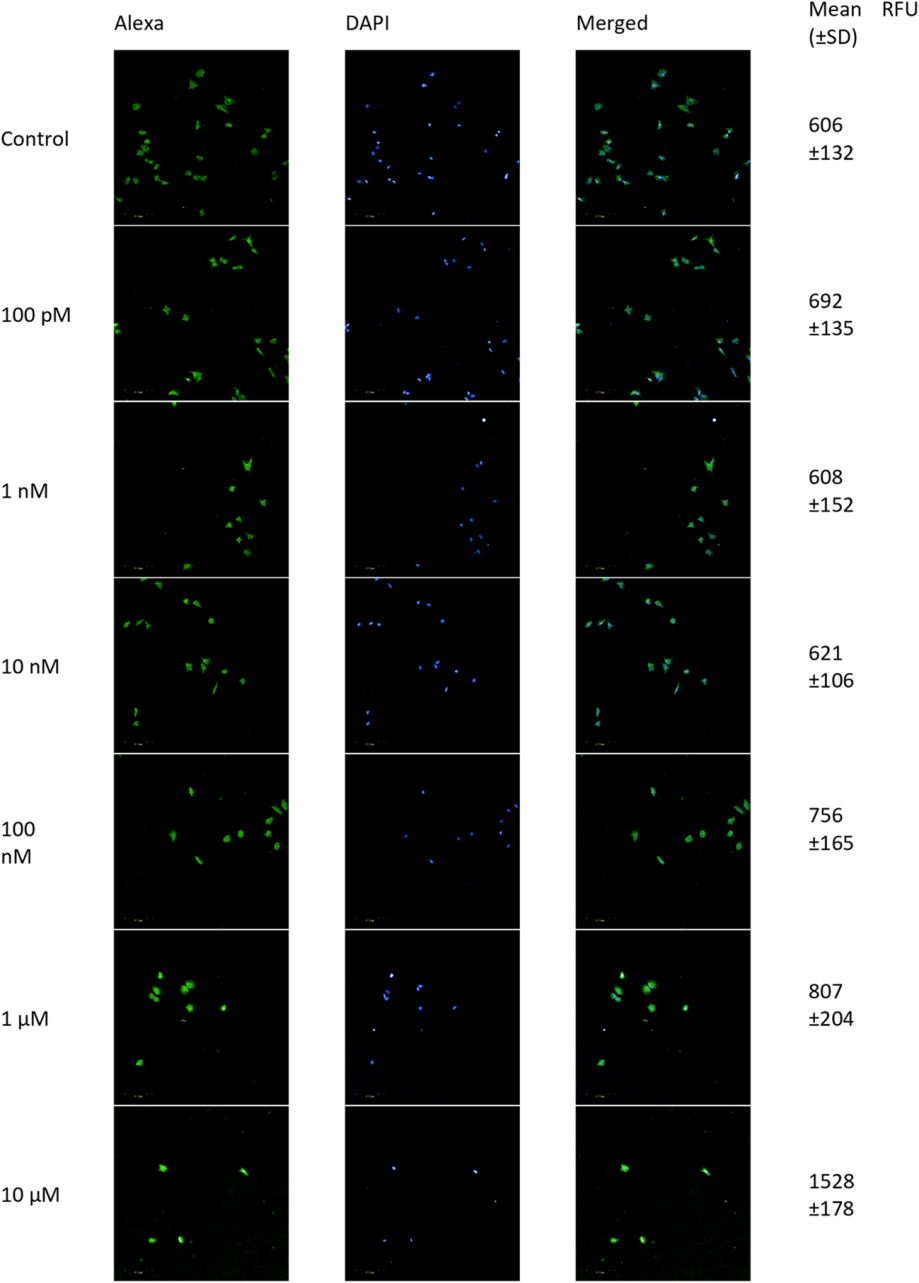
Confocal microscopy images from the TUNEL assay. Detection of apoptotic cells based on DNA fragmentation labeling in the NB SH-SY5Y cell line. FITC and DAPI staining for the nucleus. Representative images and the mean RFU values are shown

After induction of ROS and RNS, we examined if the nucleus might be targeted by 2-ME2. DNA fragmentation was analyzed by TUNEL assay. Terminal deoxynucleotide transferase (TdT) attaches FITC-labeled deoxyuridine triphosphates (FITC-dUTP) to the free 3′ ends of single or double-stranded DNA breaks for fluorescence imaging. After 24 h of treatment with 2-ME2 at a concentration range of 100 pM to 10 μM, the NB SH-SY5Y cells were tested for DNA fragmentation. Figure 4 shows representative confocal images and mean RFU values.

**Fig. 5:**
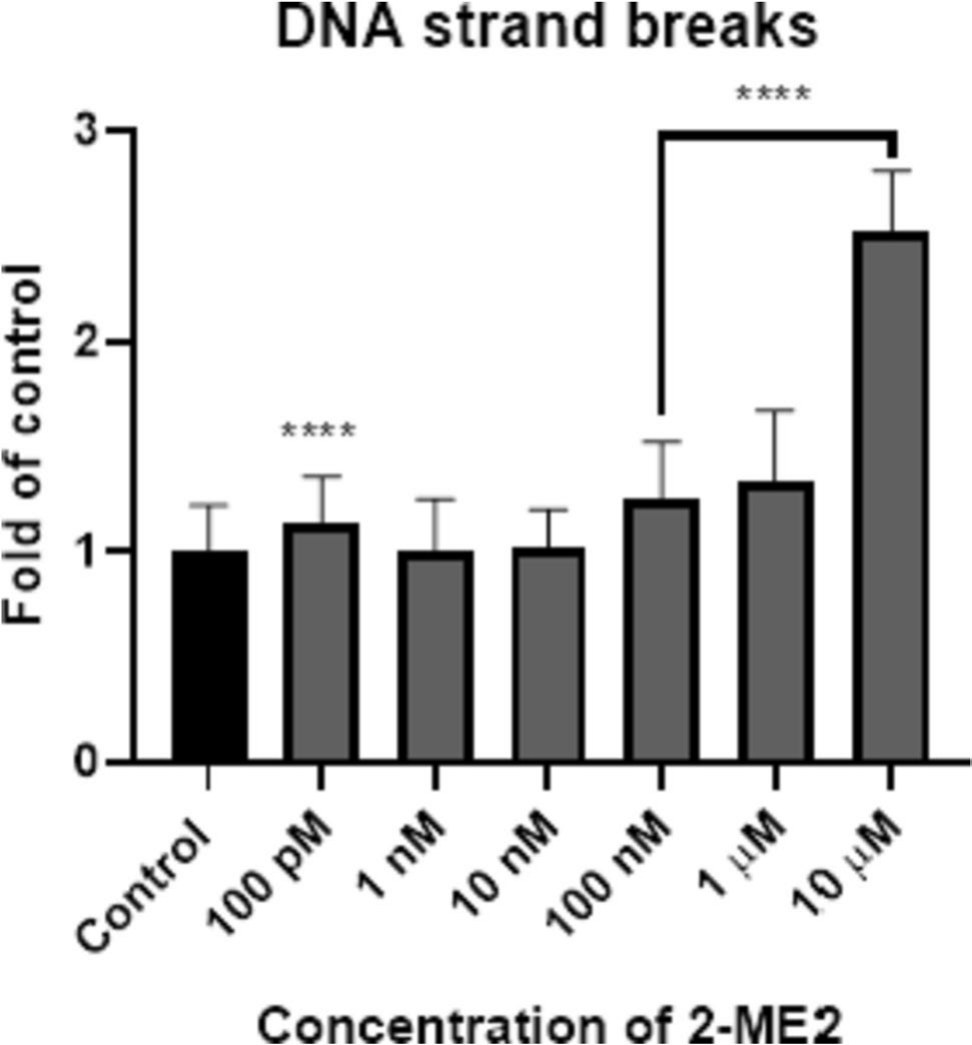
DNA strand breaks in NB SH-SY5Y cells after treatment with 100 pM–10 μM 2-ME2. Values are the mean ± SE from three independent experiments, expressed as fold change as compared to the control. The data were analyzed using GraphPad Prism, Inc., version 8, USA, by performing a one-way ANOVA with the following Dunnett's multiple comparison test against control. ****p < 0.0001 vs. control

TdT-labeled cells with DNA breaks had significantly greater RFU than controls (non-treated cells). For the NB SH-SY5Y cell line, the level of TdT-labeled cells in control (cells not treated with 2-ME2) was 1, whereas, for samples treated for 24 h with 2-ME2, the values were as follows: 100 pM–1.1 (± 0.2), 100 nM–1.2 (± 0.3), 1 µM–1.3 (± 0.3), and 10 µM–2.5 (± 0.3), relative to the control (Fig. 5).

#### 2-ME2 Effect on Nitro-oxidative Stress Level as Assessed by Western Blot Analysis

As 2-ME2 mediated an increase in the level of RNS in NB SH-SY5Y cells, a potential influence of 2-ME2 on NOS protein isoforms was studied. NOS family enzymes convert L-arginine in the presence of nicotinamide adenine dinucleotide phosphate (NADPH) and oxygen into nitrogen monoxide (NO). The levels of three NOS isoforms: nNOS, iNOS, and eNOS were studied in the NB SH-SY5Y cells using Western blot analysis. Positive controls for eNOS and iNOS were performed on Human Primary Aortic Endothelial Cells (HAEC) to exclude experimental artefacts (supplementary material).

**Fig. 6:**
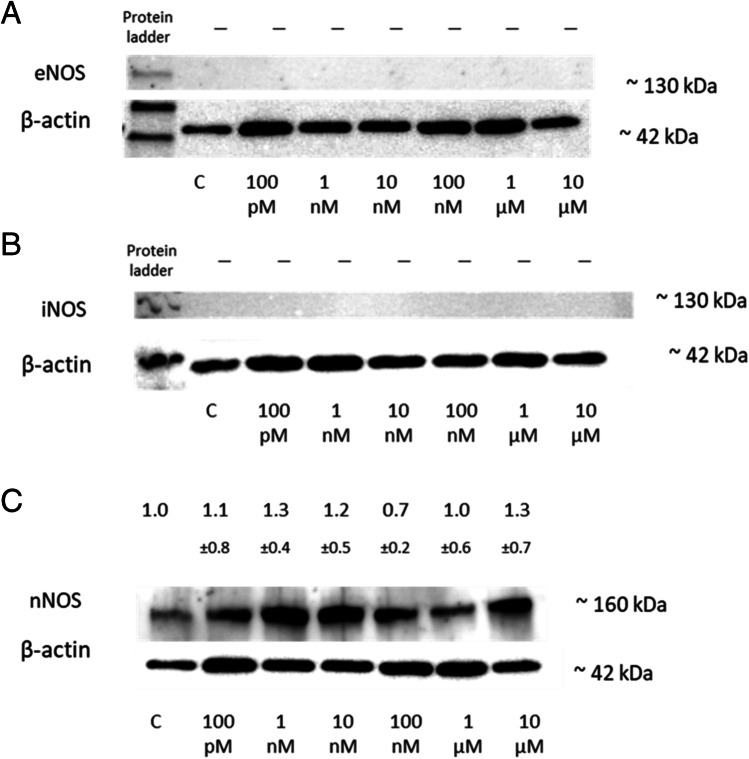
The effect of 2-ME2 on NOS level in NB SH-SY5Y cells. **A** No change in eNOS levels in NB SH-SY5Y cells treated with 100 pM–10 µM 2-ME2, as measured by Western blotting. **B** Western blot analysis reveals no change in iNOS levels in NB SH-SY5Y cells treated with 100 pM–10 µM 2-ME2. **C** The effect of 100 pM–10 µM 2-ME2 on nNOS levels in NB SH-SY5Y cells was evaluated by Western blotting. Utilizing Quantity One 4.6.6, densitometric analysis of the nNOS/β-actin ratio was conducted. The immunoblots displayed are representative of a single-membrane experimental analysis. Values are the mean ± SD from three separate experiments

24-h treatment of SH-SY5Y neuroblastoma cells with 2-ME2 at concentrations ranging from 100 M to 10 µM did not affect eNOS and iNOS cellular protein levels (Fig. 6a and b).

However, the nNOS level changed depending on the concentration of 2-ME2 used and was as follows: 1.1 (± 0.8) for 100 pM, 1.3 (± 0.4) 1 nM, 1.2 (± 0.5) 10 nM, 0.7 (± 0.2) 100 nM, 1.0 (± 0.6) 1 µM, and 1.3 (± 0.7) for 10 µM, expressed as a fold of the control level (Fig. 6c).

#### 2-ME2 Mediated Regulation of Cytochrome C Release and Heat Shock Protein Levels (HSP60 and HSP 90) in Neuronal Cells

Mitochondrial malfunction affects various interconnected cellular pathways, leading to the damage to intracellular components and the release of cytochrome C. When cytochrome C is released into the cytosol, the mitochondrial apoptosis pathway is activated, leading to apoptotic cell death [34]. Therefore, an experiment was planned to evaluate the effect of 2-ME2 on the cytosolic level of cytochrome C after 24 h of treatment of the cells.

**Fig. 7:**
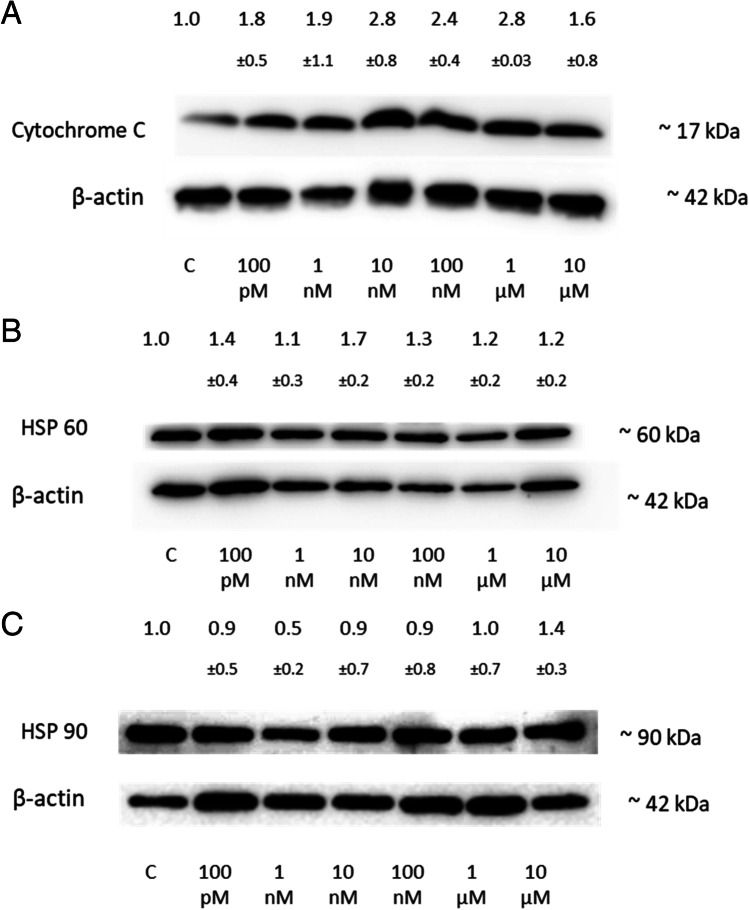
The influence of 2-ME2 on cytosolic cytochrome C level and HSP in NB SH-SY5Y cells. **A** Western blot analysis was used to determine the impact on the cytosolic level of cytochrome C by treating NB SH-SY5Y cells with 100 pM to 10 µM 2-ME2. **B** Western blotting was utilized to determine the influence of 2-ME2 concentrations ranging from 100 pM to 10 µM on HSP 60 levels in NB SH-SY5Y cells. **C** Western blotting was used to investigate the influence of 2-ME2 concentrations ranging from 100 pM to 10 µM on HSP90 levels in NB SH-SY5Y cells. Using Quantity One 4.6.6 software, densitometric analyses of the ratios cytochrome C/β-actin, HSP 60/β-actin, and HSP 90/ β -actin were conducted. The immunoblots displayed are representative of a single membrane. Values represent the mean ± SD from three independent experiments

The cytosolic cytochrome C protein levels in NB SH-SY5Y cell line measured by Western blotting after 24h incubation with 2-ME2 were as follows: 1.8 (± 0.5) for 100 pM, 1.9 (± 1.1) for 1 nM, 2.8 (± 0.8) for 10 nM, 2.4 (± 0.4) for 100 nM, 2.8 (± 0.03) for 1 µM, and for 1.6 (± 0.8) for 10 µM, expressed as a fold of the control (Fig. 7a). An increase in the cytosolic level of cytochrome C may indicate that treatment of the cells with 2-ME2 caused the release of cytochrome C from the mitochondrial intermembrane space into the cytosol.

Molecular chaperones/co-chaperones are proteins that facilitate the folding of other proteins into a specific molecular shape that is functionally active. It has been shown that chaperones and co-chaperones regulate the function of PD-related proteins by interacting with them. HSP90 and tiny heat shock proteins are known to be able to avert neurodegeneration. Understanding the crucial role of chaperones in the course of PD might help to use them as additional biomarkers for early detection of PD [35].

The changes of HSP60 protein levels in NB SH-SY5Y cell line assessed by Western blotting after 24 h incubation with 2-ME2 were as follows: 1.4 (± 0.4) for 100 pM, 1.1 (± 0.3) for 1 nM, 1.7 (± 0.2) for 10 nM, 1.3 (± 0.2) 100 nM, 1.2 (± 0.2) for 1 µM, and 1.2 (± 0.2), expressed as a fold of the control (Fig. 7b).

The changes of HSP90 protein levels in NB SH-SY5Y cell line assessed by Western blotting after 24 h incubation with 2-ME2 were as follows: 0.9 (± 0.5) for 100 pM, 0.5 (± 0.2) for 1 nM, 0.9 (± 0.7) for 10 nM, 0.9 (± 0.8) 100 nM, 1.0 (± 0.7) for 1 µM, and 1.4 (± 0.3) for 10 µM, expressed as a fold of the control (Fig. 7c).

### Clinical Study Results

#### Analysis of Plasma from Patients with PD

**Fig. 8:**
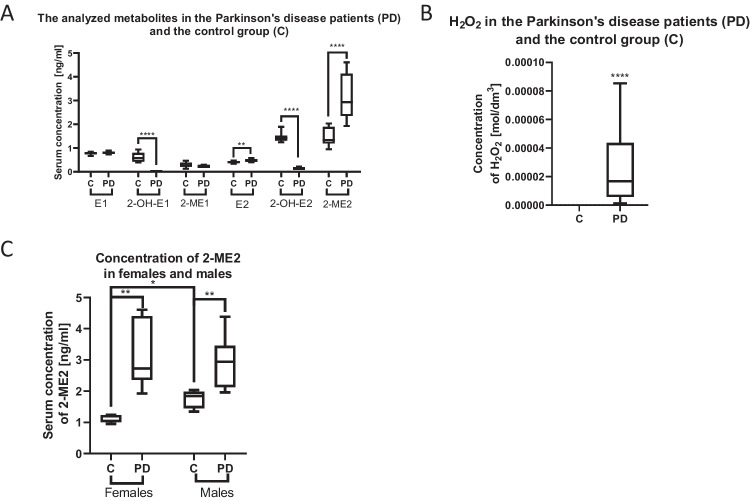
**A** Comparison of plasma estrogens and their derivatives levels in PD patients with healthy controls using LC–MS/MS analysis. Statistical analysis was performed with Mann–Whitney *U*-test. **p = 0.009, ****p < 0.001. **B** Comparison of plasma H_2_O_2_ levels in PD patients with healthy controls using stopped-flow analysis. Statistical analysis was performed with Mann–Whitney *U*-test. ****p < 0.0001. **C** Comparison of plasma 2-ME2 levels in females and males using LC–MS/MS analysis. Statistical analysis was performed with Mann–Whitney U-test. **p < 0.01 and *p < 0.1

In light of the neurotoxic activity of 2-ME2, as observed in PD in vitro model, we decided to investigate a translational value of the obtained results. The concentrations of estrogens and their selected derivatives were determined in the plasma of PD patients by the LC–MS/MS method (Table 1 and Fig. 8a).

**Table 1:** Descriptive statistics of the analyzed metabolites in the blood plasma of PD patients (1) compared to the healthy control (2) in ng/mL. Determination by LC–MS/MS. Statistical analysis was performed with Mann–Whitney *U* test. The value of *p* < 0.05 was considered statistically significant and is presented in bold

Derivative	Me		Min		Max		Q1		Q3		Statistical test result
	1	2	1	2	1	2	1	2	1	2	
E1	0.79	0.8	0.73	0.67	0.9	0.84	0.9	0.74	0.95	0.82	*U* = 60.5; *p* = 0.52
2-OH-E1	0	0.58	0	0.39	0	0.93	0	0.43	0	0.79	***U* = 0; *p* < 0.001**
2-ME1	0.23	0.25	0.2	0.13	0.3	0.46	0.3	0.22	0.24	0.39	*U* = 44.5; *p* = 0.12
E2	0.49	0.41	0.41	0.35	0.59	0.48	0.59	0.38	0.52	0.44	***U* = 26; *p* = 0.009**
2-OH-E2	0.15	1.45	0.12	1.24	0.23	1.9	0.23	1.31	0.16	1.53	***U* = 0; *p* < 0.001**
2-ME2	2.93	1.34	1.93	0.95	4.61	2.04	4.61	1.2	4.13	1.89	***U* = 4; *p* < 0.001**

Furthermore, the concentration of H_2_O_2_ was determined by the stopped-flow analytical method. The median concentration of H_2_O_2_ in the blood plasma of PD patients was 1.67 × 10^−5^mol/dm^3^ (min = 1.32 × 10^–6^, max = 8.53 × 10^–5^, Q1 = 7.23 × 10^–6^, Q3 = 3.99 × 10^–5^) while the level of H_2_O_2_ in healthy control was below the quantification threshold. i.e. below 10^–7^ mol/dm^3^ (Fig. 8b).

Additionally, the comparison of 2-ME2 levels between men and women was conducted. A statistically significant difference was found between the 2-ME2 levels of healthy women (Me = 1.2) and female PD patients (Me = 2.73) as well as between the male control group (Me = 1.85) and male PD patients (Me = 2.94). Interestingly, according to statistics, healthy males exhibit a higher concentration of 2-ME2 than healthy women (Fig. 8c).

## Discussion

### 2-ME2 Acts as a Neurotoxin Towards NB SH-SY5Y Cells

The NB SH-SY5Y cell line is exploited as a prominent in vitro model in PD research. This line is actually a subline of the SK-N-SH cells, which was established in 1970 as derived from a bone marrow biopsy of a 4-year-old girl with metastatic neuroblastoma and was subjected to three rounds of clonal selection [36]. Initial evaluation of the neuroblastoma SH-SY5Y cell line demonstrated moderate dopamine–hydroxylase activity and low levels of choline acetyl-transferase, acetyl-cholinesterase, and butyryl-cholinesterase, as well as tyrosine hydroxylase activity and nor-adrenaline (NA) release [36, 37]. Tyrosine hydroxylase transforms tyrosine to L-DOPA, the precursor of dopamine (DA), and then, dopamine–hydroxylase subsequently turns DA to NA [38]. As a consequence, the SH-SY5Y cell line has a catecholaminergic phenotype, as it possesses the enzymatic machinery to produce both DA and NA. Although these characteristics do not categorize SH-SY5Y cells as solely dopaminergic, this cell line has been regularly utilized as an experimental model for PD research [37, 39–41]. In the vast majority of published studies, retinoic acid (RA)–differentiated NB SH-SY5Y cells were not utilized. Until recently, the RA differentiation process has been used to drive the cell line toward a dopaminergenic phenotype. However, the origin and treatment of the cells may account for variance in the differentiation protocol’s outcome. In addition, the use of pharmaceutical agents in order to transform the cell line into a more dopaminergic or so to say, neuronal population may affect traits unrelated to the goal phenotype and produce ambiguous outcomes [37]. Because of the above, we decided to carry out the experiments using non-differentiated NB SH-SY5Y cells.

The route of E2 metabolism involves sequential hydroxylation and methylation [42]. The oxidation at carbon 2 within the aromatic A ring of estradiol is catalyzed by cytochrome P450 isoform 1A1 and yields 2-OH-E2 at first. The COMT enzyme, which is found in multiple organs including the brain, then replaces the hydroxyl group previously added to 2-OH-E2 with a methoxy group, resulting in a 2-ME2 molecule [42–44]. According to Zachcaria et al., 2-OH-E2 acts as a direct substrate for 2-ME2 synthesis. However, cell treatment with 2-OH-E2 does not necessarily have similar effects to the treatment with 2-ME2. The reason for that is the possibility of an alternative intracellular transformation of 2-OH-E2 into quinine derivatives of high reactivity in the biological system. 2-ME2 is metabolically rather inert; however, it has been proven to have various interesting biological activities, among others protecting the cell against carcinogenesis. That is why effective endogenous methylation of 2-OH-E2 to 2-ME2 is so critical for proper cellular metabolism and survival [45].

In our research model, concentrations corresponding to the physiological and pharmacological levels of 2-ME2 were used. After 24 h treatment of the NB SH-SY5 line, a decrease in cell viability was observed only at 2-ME2 concentrations corresponding to the pharmacological range, which has been previously confirmed by our team in a similar experimental model [12]. Zhang et al. also demonstrated decreased viability of SH-SY5Y cells after exposure to 2-ME2 [46].

One of the cytostatic mechanisms of action of 2-ME2 in the experimental models is the arrest of cells in the subG1 phase, which suggests the induction of apoptosis. Additionally, 2-ME2 was also found to increase the cell quantity in the subG1 phase in other neural cells [11, 47]. The increase in the number of apoptotic cells noted across a panel of concentrations corresponding to both pharmacological and physiological levels was observed. There is little research into the induction of apoptosis by 2-ME2 in neural, healthy, or neoplastic cells. Our team previously demonstrated the induction of apoptosis in SH-SY5Y cells [12] and in mouse HT22 hippocampal cells [9]. Also. previously mentioned Zhang et al. showed an increase in the number of apoptotic cells in the SH-SY5Y line incubated with pharmacological levels of 2-ME2 [46]. Moreover, 2-ME2 was proven to induce apoptosis in cellular models of tumors such as melanoma [48], osteosarcoma [9, 10], prostate [49–51], or breast cancer [52–54]. In addition, as observed in the in vivo studies, 2-ME2 did not protect hilar hippocampal neurons from the excitotoxicity induced by kainic acid [55].

Multiple interrelated cellular pathways are affected by mitochondrial dysfunction, resulting in intracellular component damage and the release of cytochrome C. When cytochrome C is released, the mitochondrial apoptosis pathway is initiated, resulting in apoptosis. The results indicated a rise in the level of cytochrome C, which may suggest that cytochrome C was released from the mitochondria into the cytosol due to 2-ME2 activity. Moreover, 2-ME2 was found to stimulate cytochrome C release in many cancer cells including prostate cancer cells [56], pancreatic cancer cells [57], human chondrosarcoma cells [58], human acute T lymphoblastic leukemia CEM cells [59], and fibroblasts [60]. The above-mentioned studies clearly indicate that the intrinsic apoptotic pathway is involved in 2-ME2-dependent apoptosis.

### 2-ME2 Induces Nitro-oxidative Stress in NB SH-SY5Y Cells

The induction of oxidative stress is a factor common to the development of both neurodegeneration and cancer [61–63], and one of the mechanisms of 2-ME2 is the induction of ROS and RNS in many cell lines [9, 11, 12, 46, 64–68]. In the above study, it was revealed that in NB SH-SY5Y cells treated with 2-ME2, the levels of ROS and RNS at concentrations corresponding to pharmacological levels were increased. With the use of fluorophotometric analysis, induction of ROS in the SH-SY5Y line was detected within the range of pharmacological concentrations [46]. In the case of RNS induction, at physiologically and pharmacologically relevant concentrations of 2-ME2, elevated levels of NO were determined [12].

HSP90 inhibits the aggregation of α-synuclein (α-syn) in an in vitro experiment [69]. Moreover, inhibiting HSP90 decreased oligomeric α-syn, which in turn increased dopamine in PD [70]. In a cellular model of PD, the HSP90 inhibitor, geldanamycin, was reported to diminish the production of α-syn aggregates and α-syn-induced toxicity [71]. After inducing apoptosis in NB SH-SY5Y cells, Hsp90 enhances the cell survival rates [72]. In the present study, for the first time, we have demonstrated 2-ME2—depended increased level of HSP 90 in NB SH-SY5Y cells. Previously, 2-ME2 was found to induce HSP90 in cancer cell lines such as osteosarcoma 143B [73], or breast cancer MCF-7 [74]. In addition, mitochondrial toxin 1-methyl-4-phenylpyridinium (MPP +), the most effective and widely used toxin for the development of an in vitro PD model, also induced HSP 90 in SH-SY5Y cells [75]. Based on the above, inhibiting HSP90 could be a possible treatment method or preventive measure against PD [35].

The obtained 2-ME2-mediated induction of HSP 90 correlates with the elevated level of nNOS in NB SH-SY5Y cells. It is suggested that Hsp90 directly enhances nNOS-catalyzed NO synthesis, which is largely dependent on the augmentation of calmodulin binding to nNOS. In addition, Hsp90 is necessary for heme binding and the production of catalytically active nNOS [76, 77]. 2-ME2 was found to induce nNOS in not only cancerous cell lines such as osteosarcoma 143B [9, 10, 14], and MG63.2 [73], glioblastoma SW1088 [31], but also in mouse hippocampal HT22 cells [9], leading to the elevation of NO and a resultant cell death [9]. It is quite appealing that 2-ME2 induces only nNOS, while it does not affect iNOS nor eNOS. In accordance with the study presented above, neither of these isoforms was induced by 2-ME2 in both osteosarcoma and melanoma cell models [9, 78]. Unlike eNOS and iNOS, nNOS is a larger protein with a PDZ domain at its N-terminus, a consensus sequence of about 90 amino acids [79]. PDZ domain proteins are essential for cellular migration and ion channel surface retention. They can also function as scaffolds for the recruitment of structural and regulatory components to the cell membrane [80]. The domain appears to be a key element in nNOS transport to various intercellular compartments [79]. PDZ is required for nuclear recruitment of nNOS, hence favoring NO production [81], and furthermore, PDZ proteins are required for neural signaling [80]. Local NO production and its reactive byproducts such as nitrogen dioxide and peroxynitrite, are most likely contributing to DNA damage [82, 83]. We previously demonstrated that the generation of NO derivatives is at least primarily reliant on selective nNOS overexpression and plays a crucial role in the 2-ME2-mediated cell death mechanism [14].

HSP60 is a cytoplasmic and mitochondrial protein engaged in the folding, refolding, transportation, and translocation of proteins. Depending on its cellular location, HSP 60 can play both pro- and anti-apoptotic roles. Furthermore, central nervous system (CNS) damage also results in the extracellular release of HSP 60, which activates microglia and subsequently the innate immune system. Current data indicate that inflammatory responses expressed by glial reactions are widely regarded as significant aspects of PD [84]; therefore, the HSP 60 level in NB SH-SY5Y cells after incubation with 2-ME2 was analyzed.

In the above studies, an increase in the level of HSP 60 in NB SH-SY5Y cells under the influence of 2-ME2 at both physiological and pharmacological concentrations was demonstrated. Elevated HSP 60 level was also observed in another PD cellular model, i.e., in PC12 cells treated with 6-hydroxydopamine in order to induce cellular degradation [84]. Previously, our team has also confirmed the induction of HSP60 in SW1088 grade III glioma cells [31]. HSP 60 can be released by CNS cells experiencing necrotic or apoptotic cell death in order to activate microglia [85].

DNA damage occurs in the neuronal genome, most likely as a result of significant oxidative stress in the brain [86–88]. Additionally, we previously demonstrated that RNS produced by 2-ME2 are DNA damage inducers due to their affinity for the guanine base of DNA [14]. Herein, 2-ME2-mediated DNA strand breaks of NB-SH-SY5Ycells were demonstrated. So far 2-ME2 was found to induce DNA impairment in osteosarcoma 143B cells [10] and SW1088 grade III glioma cells [31].

### Estradiols and H_2_O_2_ as Fast and Economical Tool for PD Diagnostics at the Early Stage of the Disease

Behl et al. demonstrated that E2 and certain estradiol derivatives can prevent intracellular H_2_O_2_ accumulation and, eventually, degeneration of primary neurons, clonal hippocampal cell, and cells in the organotypic hippocampus. Interestingly, the neuroprotective antioxidant effect of estrogens depends on the presence of the hydroxyl group at the C3 position on the A ring of the steroid molecule, but is independent of estrogen receptor activation [89]. However, only clinical studies carried out in the course of work on this project, allowed to determine the relationship between the concentration of estrogens and their selected derivatives: hydroxy and methoxy estrogens, correlating with the induction of H_2_O_2_ and their role in the diagnosis of PD.

The determination of 2-OH-E2 and more importantly, 2-ME2, may be applied as biomarkers for the diagnosis of the neurodegenerative process in PD. It is unclear whether there are similar relationships in other neurodegenerations, so it definitely requires further research. In addition, the in the plasma of people with PD much higher concentrations of H_2_O_2_ were measured in comparison to healthy volunteers. The studied samples obtained from patients showed a reduced level of hydroxylated estradiol derivatives in favor of methylated derivatives and a high level of H_2_O_2_. The above data shows that the decreased level of the above-mentioned hydroxyestardiols and the increased level of the 2-ME2, correlating with the level of H_2_O_2_ can serve as a biomarker for the diagnosis of PD. Furthermore, as we previously indicated, 2-ME2 may serve as a diagnostic and tracking biomarker in lung cancer patients [32]. Similar to our results, the reduced excretion of 2-ME2 in the urine of endometrial cancer patients was observed by Zhao et al. [90]. The findings suggest that the metabolic pathway of estrogens is linked to carcinogenesis and may give useful biomarkers, such as 2-ME2, for assessing the risk of estrogen-induced breast cancer [90]. Both studies determined decreased level of 2-ME2 in cancerous patients [32, 90], which may indicate that the methylation pathway may play a significant role in cancers [90]. Interestingly, the research of Pérez-Sepúlveda et al. demonstrate that 2-ME2 levels in early pregnancy may also be useful to foresee the eventual development of preeclampsia (PE), as plasma 2-ME2 levels were lower in women who subsequently had PE at 11 to 14 weeks of pregnancy [91, 92]. On the other hand, endogenous 2-ME2 blood levels in malignant melanoma (MM) are not effective as a diagnostic or prognostic indication as there was no connection between 2-ME2 serum levels and early or advanced-stage illness in individuals with MM [93]. Here, for the first time, a 2-ME2 serum concentration was established in neurological disease, and in contrast to cancers and PE, its levels are elevated. More specifically, the results obtained on the SH-SY5Y cell line are confirmed by the outcome of the patients. From the above, it can be concluded that 2-ME2 may play its own role in neurodegeneration.

What is more, the obtained results clearly suggest a higher level of 2-ME2 in healthy men in comparison to women. According to epidemiological data, men more often suffer from PD than women [94, 95]. Our findings may explain a possible susceptibility of men to PD development. Clearly, additional research is necessary to determine the default 2-ME2 level in men, as well as to determine if this level varies with age and if it is associated with the future development of PD. Intriguingly, studies have already demonstrated a correlation between the incidence of PD in men and milk consumption [96, 97].

## Conclusions

The pathways leading to the development of neurodegenerative disease and the death of cancer cells may overlap [20, 21]. The main factors of neurodegeneration are suggested to play a key role in regulating tumor growth [63]. The PD patients show some resistance towards cancer [20, 21, 98], and this phenomenon actually drew the authors’ attention to the problem of neurodegeneration pathomechanism and lower cancer incidence as observed in the course of PD [99].

2-ME2 is known for its anticancer activity [100–103]; however, there are data suggesting its neurotoxicity [11, 99]. Herein, the cytotoxic activity of 2-ME2 toward the NB SH-SY5Y cell line in the neurodegeneration cellular model was demonstrated. 2-ME2 induced apoptosis in NB SH-SY5Y cells by cytochrome C release and regulation of HSP60. Moreover, 2-ME2 generated ROS and RNS in NB SH-SY5Y which resulted in DNA breakage. The increased level of HSP90 and HSP90-regulated nNOS [104] were determined and proved to be responsible for oxidative stress generation.

Subsequently, for the first time, we verified the plasma levels of estrogens and their derivatives in PD patients to provide a translational relevance for our study. The obtained results clearly indicate an increased level of 2-ME2 and H_2_O_2_ in the blood of patients with a simultaneous decrease in the level of 2-OHE2. We observed an inverse relationship between 2-OHE2 and 2-ME2 concentrations, which may suggest rapid metabolism of 2-OHE2 to 2-ME2 and simultaneous oxidative stress generation. Our results on the NB SH-SY5Y cell line, which is an in in vitro model for PD, clearly indicate that 2-ME2 induces, in particular, nitro-oxidative stress, which leads to cell death. Moreover, in the plasma of PD patients, 2-ME2 is in higher concentrations than in healthy controls. Such results suggest that 2-ME2 may be the cause of neuronal damage in these patients, which underlies the development of PD. Therefore, we suggest that elevated concentrations of 2-ME2, as a neurotoxic metabolite of E2 naturally occurring in the body, may represent a biomarker for PD.

Definitely, more extended research is required on a larger group of patients and also in other forms of Parkinsonism. Because the participants in the above study had H&Y scores II or III, determining the level of 2-ME2 in early PD and the link with PD progression is impractical to solve. As a result, we are undertaking additional analyses on a larger sample of patients to investigate the connection between disease progression and the level of 2-ME2 in the early stages of PD H&Y score I. An analysis of a larger number of patients with PD in the very early or late stage of the disease will allow us to assess the diagnostic potential of the novel biomarkers which we are proposing. Another limitation of the presented research is the lack of data pertaining to other neurodegenerations and potential reflection of the intracellular neurodegenerative processes in general. Furthermore, the presence of patients with advanced stages of the disease would help to answer the question of whether it is an increase in methoxy estrogens has a linear character over time. Moreover, the group of premotor phase patients, e.g., with RBD (REM-sleep behavior syndrome) would be required which is by far the most sensitive clinical biomarker of the prodromal phase of synucleinopathies.

Based on the above, we suggest that plasma hydroxy and methoxy estrogens, as well as H_2_O_2_, may be applied as clinical biomarkers for PD diagnosis and treatment. We strongly believe that our results will provide a widely available PD diagnostic tool for relatively easily identifying neurodegeneration symptoms, which would enable to diagnose patients at an early stage of PD and allow early treatment with modern drugs. Moreover, as we have previously stated, 2-ME2 may act as a diagnostic and monitoring biomarker in patients with lung cancer [32].Here, for the first time in neurological disease, a 2-ME2 serum concentration was determined. Notably, the clinical application of our findings would not pose much of a challenge, as it primarily entails the determination of specific estrogen metabolites in patients' blood making it a practical minimally invasive diagnostic method that is also relatively fast and inexpensive. The described research is the subject of the patent application “The use of hydrogen peroxide and 17β-estradiol and its metabolites as biomarkers in the in vitro diagnosis of neurodegenerative diseases,” no. P.441360.

### Supplementary Information

Below is the link to the electronic supplementary material.

Supplementary file1 (DOCX 81 KB)

## Data Availability

The data used to support the findings of this study are available from the corresponding authors upon request.
