# Supplementary material for: 2-Methoxyestradiol and Hydrogen Peroxide as Promising Biomarkers in Parkinson’s Disease
Source: Mol Neurobiol. 2023 Aug 17;61(1):148–66. doi: 10.1007/s12035-023-03575-6 (PMC10791893; doi:10.1007/s12035-023-03575-6)
Supplement: Supplementary file 1 — Supplementary file1 (DOCX 81 KB) [file 12035_2023_3575_MOESM1_ESM.docx]

**Suplementary Materials**


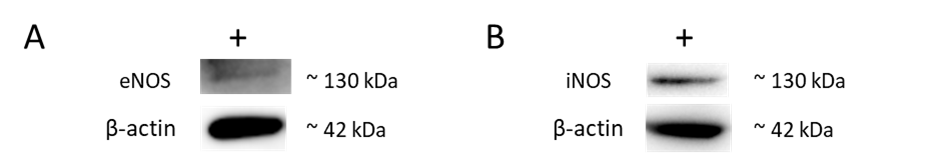


Positive controls for eNOS (A) and iNOS (B) on Human Aortic Endothelial Cells (HAEC) by western blot.
